# Associations between self-reported vegetable and fruit intake assessed with a new web-based 24-h dietary recall and serum carotenoids in free-living adults: a relative validation study

**DOI:** 10.1017/jns.2019.23

**Published:** 2019-08-05

**Authors:** J. Lafrenière, C. Couillard, B. Lamarche, C. Laramée, M. C. Vohl, S. Lemieux

**Affiliations:** Institute of Nutrition and Functional Foods, School of Nutrition, Laval University, Québec, QC, Canada

**Keywords:** Carotenoids, Vegetables and fruit, Food assessment, 24-h dietary recall, Validation, FM, fat mass, R24W, web-based 24-h dietary recall, VF, vegetable and fruit, WC, waist circumference

## Abstract

The aim of the present study was to assess the relative validity of a new web-based 24-h dietary recall (R24W) in terms of vegetable and fruit (VF) intake assessment using serum carotenoid concentrations as reference biomarkers. A total of seventy-four women and seventy-three men (mean age 47·5 (sd 13·3) years; mean BMI 25·5 (sd 4·4) kg/m^2^) completed the R24W four times to assess their VF intake. Serum carotenoids were obtained from 12-h fasted blood samples and measured by HPLC. Raw and de-attenuated partial Spearman's correlations were performed to determine how usual vegetable and/or fruit intake was associated with serum carotenoids. Relevant confounders were selected using a stepwise regression analysis. Finally, cross-classification was used to determine agreement between intake of VF and serum carotenoids. Intake of total dietary carotenoids was significantly associated (*r* 0·40; *P* < 0·01) with total serum carotenoids (without lycopene). Total VF intake was also associated with total serum carotenoid concentrations without lycopene (*r* 0·44; *P* < 0·01). HDL-cholesterol, waist circumference and age were identified as confounders in the association between total VF intake and total serum carotenoids (without lycopene). De-attenuated partial correlation adjusted for these confounders increased the associations between dietary carotenoids and total serum carotenoids without lycopene (*r* 0·49; *P* < 0·01) and between total VF intake and total serum carotenoids without lycopene (*r* 0·48; *P* < 0·01). Almost 80 % of respondents were classified in the same or the adjacent quartile for total VF intake and total serum carotenoids without lycopene, while less than 6 % were classified in the opposite quartile. Overall, these observations support the appropriateness of the R24W to assess the dietary intake of VF.

Increasing the population's vegetable and fruit (VF) intake is one of the main nutritional objectives of public health organisations^(1)^. There is significant evidence that consumption of VF is associated in a dose-dependent manner with better cardiovascular health^(2)^ and a lower risk of all-cause mortality^(3)^. Getting an adequate amount of VF is also essential to reaching dietary reference intakes of most of the important vitamins and minerals^(4)^. In the 2007 edition of Canada's Food Guide, specific advice has been added to encourage Canadians to eat more dark green and orange VF in order to optimise their nutrient intake^(5)^. To track positive outcomes of these recommendations, we must rely on self-assessed dietary assessment. This approach is useful to analyse dietary patterns or nutrient intake at the population level. However, it is also associated with misreporting as well as with suboptimal accuracy and reproducibility at the individual level^(6,7)^. Therefore, many researchers are working on strategies to improve the objectivity of dietary assessment's measuring tools.

According to different reviews^(8,9)^, serum carotenoid concentrations appear to be the best blood marker of VF given that they are primarily found in plants and that they cannot be synthesised by the human body^(10)^. Up to now, more than 600 species of carotenoids have been discovered and about sixty of them have already been measured in human serum^(11)^. The six most common are α-carotene, β-carotene, β-cryptoxanthin, lycopene, lutein and zeaxanthin^(12)^. They are characterised by a chemical structure containing a conjugated double bound that gives plants their yellow or orange colour, although this colour is sometimes masked by a stronger pigment, that is, the chlorophyll in dark green vegetables^(13)^. Serum carotenoids can be classified as concentration biomarkers as opposed to recovery biomarkers, which can be directly linked to tissue concentration^(14)^. Up to now, besides doubly labelled water (for energy expenditure), 24-h urinary nitrogen (for protein intake) and K, very few recovery biomarkers have been implemented in nutritional research. Concentration biomarkers are also associated with intakes but with a larger between-individual variability caused by differences in absorption and metabolism^(15)^.

The main challenge facing researchers who are trying to use carotenoids as biomarkers is the lack of a proportional relationship between dietary intake and serum levels. Indeed, VF contain varying amounts of different carotenoids (see Table 1). Moreover, their concentration differs from one vegetable or fruit to the next^(16)^. As an example, it has been shown that broccoli and green peas are more effective than spinach (in mg of carotenoids consumed) in increasing serum lutein levels^(17)^. From these observations, it seems clear that it would be impossible to characterise one's VF intake with a specific carotenoid. It is also the conclusion reached by Baldrick *et al*.^(12)^ in a review about the effect of different VF on serum carotenoid levels. They stated that the best strategy for assessing total VF intake was to use a panel of carotenoids.

**Table 1.** Food sources of the most common carotenoids*

Carotenoids	Food sources
α-Carotene and β-carotene	Orange fruit and vegetables, dark green vegetables such as spinach, peas, green beans
β-Cryptoxanthin	Oranges, papayas, nectarines
Lutein and zeaxanthin	Spinach, broccoli, maize, apricots, nectarines
Lycopene	Tomatoes and food derived from tomatoes

* Adapted from: Breithaupt & Bamedi^(46)^, Jansen *et al*.^(10)^, Rodriguez-Amaya^(47)^.

Besides using multiple serum carotenoids, adjusting for some factors involved in their metabolism is essential to get an adequate biomarker of VF. Using data from 264 adults put on a diet containing between six and twenty-one servings of VF per d, Couillard *et al*.^(18)^ highlighted that, for the same intake of VF, women had higher serum levels of carotenoids. Building on this work, Allore *et al*.^(19)^ demonstrated that HDL-cholesterol mediates the sex difference in serum carotenoids potentially because of the difference in lipid density of the lipoprotein between men and women^(20)^. They also demonstrated that greater body weight and greater waist circumference (WC) were associated with lower serum levels of carotenoids. Souverein *et al*.^(21)^ used a similar approach to develop a mathematical model to predict VF intake from serum carotenoids, folate, vitamin C, and other factors such as age, BMI and sex. The model better predicted VF intake than any of the individual biomarkers, emphasising the importance of including confounders when assessing the association between VF intake and serum biomarkers.

Recently, we have developed a new web-based 24-h dietary recall (R24W) facilitating food assessment in the French-speaking population of the province of Quebec^(22–25)^. The present study aims at assessing the relative validity of the R24W to estimate VF intake using carotenoids as concentration biomarkers. We hypothesise, based on the recent literature, that VF intake is significantly associated with serum carotenoid levels and that adjusting this association for relevant metabolic confounders increases its strength.

## Experimental methods

### Participants

A total of seventy-five women and seventy-five men aged between 18 and 68 years from the Quebec City area volunteered to take part in this validation study. Exclusion criteria were pregnancy, lactation and digestive problems causing malabsorption in order to avoid any interaction in the analysis of blood biomarkers. The present study was conducted according to the guidelines laid down in the Declaration of Helsinki and all procedures involving human subjects were approved by the of the Laval University Ethics Committee. Written informed consent was obtained from all subjects.

### Sample size calculation

The present analysis is based on data from a validation study for new web-based questionnaires including the R24W. Based on previous data^(26)^, a sample size of 150 was calculated to provide 80 % power at a significance level of 0·05 to distinguish a 10 % difference in energy intake using different dietary assessment methods and to ensure adequate participant:item ratio in the questionnaire validations^(27)^. This calculation also exceeds the recommendations of the EURopean micronutrient RECommendations Aligned Network of Excellence (EURRECA) for the number of individuals who should be included in a validation study that includes the analysis of biomarkers^(28)^.

### Study protocol

Participants were invited to an initial visit at the research institute where their body height (SECA height rod Model 216; SECA Corp.), weight and body composition (TANITA body composition analyser BC-418; Tanita Corp.) were assessed. Then, the WC measurement was taken at the end of a normal expiration with a tape placed horizontally directly on the skin at the mid-distance between the last rib and the top of the iliac crest. WC was determined as the mean of three measurements to the nearest 0·1 cm. During this same visit, fasting blood samples (12-h fast) were collected from an antecubital vein into vacutainer tubes for the measurement of fasting blood lipids and carotenoids. Samples were then immediately centrifuged at 17°C for 10 min at 1100  ***g*** and stored at −80°C until processed. Blood lipid concentrations were assessed with the use of a Roche Modular P system (Roche Diagnostics).

### Serum carotenoids analysis

Details about standards preparation are described in detail in a previous study conducted in our research facility^(18)^. Carotenoid identification was performed with HPLC. Serum samples kept at −80°C were thawed in the freezer at the end of the day, a day before the analysis. Samples were vortexed and then centrifuged at 1370 ***g*** for 10 min at 4°C. Aliquots of 100 µl of serum were then transferred to Eppendorf tubes (1·5 ml) along with 20 µl of 2-propanol and 20 µl of internal standard and the tubes were vortexed. Samples were transferred on a 400 µl fixed well plate (ISOLUTE^®^ SLE+; Biotage), then there was a wait of 5 min. Then, 900 µl of hexane–isopropanol (90/10, v/v) were added twice to each well. Each extracted sample was evaporated under N_2_ and, once dried, was reconstituted with 300 µl of methanol–dichloromethane (65/35, v/v). Plates were shaken for 1 min, twice, and samples were transferred into HPLC glass vials to be analysed.

HPLC analysis of the samples was performed using an Agilent 1260 Infinity system (Agilent) equipped with a binary pump system and a C30 reversed phase column (YMC America Inc.) kept at a constant temperature (35°C). Carotenoids of the different samples were separated with a mobile phase consisting of methanol–water (98/2, v/v; Eluent A) and methyl-*tert*-butyl ether (MTBE; Eluent B; VWR). A UV detector was set at 450 nm and identification of each compound was confirmed using retention time and UV spectra (190–640 nm) of the pure compounds. Data acquisition was carried out with Chemstation software (Agilent). CV for each carotenoid was tested in our laboratory facilities using split samples and it varied between 3·68 and 10·03 % (detailed data available in Supplementary Table S1).

### Dietary intake assessment

Following the initial visit during which blood sampling for carotenoid measurement was taken, participants received emails on random unannounced days inviting them to complete the R24W four times during a period varying from 5 to 20 d between April and July 2015. In order to complete their first 24-h dietary recall, all participants had to watch a tutorial video describing all main aspects of the software^(22)^. If participants did not complete the 24-h recall on the day they received the email, the access was cancelled, and another email was sent on another unannounced day. The first dietary recall was sent 10 d after the initial visit when the blood sampling was collected. About 60 % of the cohort completed all the four 24-h recalls when they received them the first time while the other 40 % of the cohort did not fill at least one 24-h recall when they received it the first time. Overall, an interval of 15 to 30 d between blood sampling and dietary recall completion was observed.

This fully automatic software was inspired by the automated multiple-pass method developed by the United States Department of Agriculture for national dietary surveys^(29)^. A total of 2865 different food items and 687 recipes are available in the R24W^(22)^. Respondents are guided to recall their previous day's intake, meal by meal. Pictures of up to eight portion sizes are proposed for each food item described by unit and/or volume. Dietary intakes were estimated by the average of the 4 d of dietary recalls. The R24W automatically provided Canada's Food Guide servings for total VF, fruit, whole fruit, vegetables, orange vegetables and dark green vegetables. This includes servings from single foods as well as recipes. One hundred percent fruit juices were considered in the total VF and fruit categories but were not included in the whole fruit category. It should also be noted that, accordingly to Canada's Food Guide, some orange-coloured fruit such as apricot and mango are included in the total number of orange vegetables^(5)^. Energy and dietary carotenoid intakes were also automatically computed by the R24W using data from the Canadian Nutrient File database (Health Canada, 2010). The percentage of foods containing dietary carotenoids for which a value is available in the R24W ranges between 78·6 % (lycopene) and 94·4 % (β-carotene). Dietary supplement use was assessed through a web-based FFQ^(26)^ in which questions about different types of supplements were asked. Information about consumption of multivitamins and carotenoids (β-carotene) only was kept for this analysis.

### Statistical analysis

Normality of the distribution of dietary intakes and serum carotenoids was tested using the Kolmogorov–Smirnov test. As data were mostly not normally distributed, differences in dietary intake and serum carotenoids between men and women as well as between supplement users and non-users were assessed with the Mann–Whitney *U* test. For these variables, data are presented as medians and interquartile ranges. Spearman's correlations were used to assess the associations of dietary carotenoids, categories of VF (servings/d of total VF, fruit, whole fruit, vegetables, orange vegetables and dark green vegetables), age, adiposity metrics (BMI, WC and fat mass (FM)) as well as TAG, total cholesterol, LDL-cholesterol and HDL-cholesterol with serum carotenoids. As serum lycopene demonstrated a different pattern of association compared with the other serum carotenoids, data for total serum carotenoids were presented while including or excluding lycopene^(30)^. A stepwise regression analysis was used to determine the relevant confounders of the association between total serum carotenoid concentrations without lycopene and total VF intake. Serum TAG, total cholesterol, LDL-cholesterol, HDL-cholesterol, WC, body weight, FM, BMI, age, sex, intake of supplements and total energy intake were considered in this analysis. De-attenuated partial Spearman's correlations were used to assess the associations between dietary and serum carotenoids as well as between VF intake (servings/d) and serum carotenoids adjusted for significant confounders found with the stepwise regression analysis. De-attenuation of correlations adjusts for day-to-day variability among the 4 d of 24-h dietary recall^(31)^. Serum carotenoids were then compared across quartiles of total VF intake using cross-classification and the κ statistic. We conducted this analysis twice. We first performed the analysis with raw total serum carotenoids. We then performed the analysis using a composite variable derived from the stepwise regression model in order to take into consideration the significant confounders of the association between VF intake and serum carotenoids. Statistical analyses were conducted with SAS version 9.4 software (SAS Institute Inc.).

## Results

Participants' characteristics are presented in Table 2. A total of 147 participants from the 150 who were recruited were included in the analysis. Three participants were excluded because they did not complete all four 24-h dietary recalls and did not provide the blood sampling. There was no difference between women and men in age, TAG, LDL-cholesterol and BMI, whereas men had higher WC and total energy intake than women. Conversely, women had higher FM and higher concentrations of HDL-cholesterol and total cholesterol. Intake of the different categories of VF did not differ between sexes except for whole fruit intake which was higher in women than in men. Likewise, serum concentrations of β-carotene, β-cryptoxanthin and total serum carotenoids were higher in women whereas serum concentration of lycopene was higher in men. Serum concentration of carotenoids did not differ between supplement users and non-users except for the concentration of lycopene which was slightly higher in supplement users. Therefore, subsequent analyses were not adjusted for the use of supplements.

**Table 2.** Participants' characteristics (*n* 147) (Mean values and standard deviations; medians and interquartile ranges (IQR))

	Women (*n* 74)				Men (*n* 73)				
	Mean	sd	Median	IQR	Mean	sd	Median	IQR	Difference (*P*)*
Age (years)	49·8	11·5			45·2	14·5			0·07
BMI (kg/m^2^)	25·0	4·4			26·0	4·4			0·10
Waist circumference (cm)	84·6	11·8			93·7	13·5			<0·01
Fat mass (kg)	22·3	8·7			18·0	9·5			<0·01
TAG (mmol/l)	1·3	0·7			1·3	0·7			0·84
Total cholesterol (mmol/l)	5·6	1·2			5·0	0·8			<0·01
LDL-cholesterol (mmol/l)	3·1	1·0			2·9	0·7			0·15
HDL-cholesterol (mmol/l)	1·9	0·5			1·5	0·4			<0·01
Energy intake									<0·01
kJ/d	8965·1	1805·0			12 470·0	3504·9			
kcal/d	2142·7	431·4			2980·4	837·7			
Use of supplement (%)†	21·6				19·2				0·71
Vegetable and fruit intakes (servings/d)									
Total vegetables and fruit			6·1	3·1			5·7	3·3	0·87
Fruit			2·5	1·7			2·2	2·6	0·67
Whole fruit			1·6	1·1			1·1	1·3	<0·01
Vegetables			3·3	1·9			3·6	2·2	0·69
Dark green vegetables			0·8	0·8			0·6	0·8	0·07
Orange vegetables			0·3	0·4			0·3	0·6	0·44
Serum carotenoids (μmol/l)									
Serum α-carotene			0·37	0·29			0·31	0·31	0·06
Serum β-carotene			0·98	0·74			0·70	0·70	0·01
Serum β-cryptoxanthin			0·30	0·22			0·21	0·11	<0·01
Serum lycopene			0·61	0·34			0·69	0·32	0·03
Serum lutein			0·40	0·17			0·33	0·17	0·05
Serum zeaxanthin			0·11	0·06			0·11	0·05	0·11
Total serum carotenoids			2·96	1·22			2·48	1·36	0·03

* *P* value based on Mann–Whitney *U* test or χ^2^.

† Supplements considered were multivitamin and carotenoid supplements (β-carotene).

Univariate correlations between dietary carotenoids, categories of VF intake, age, anthropometric measures as well as blood lipid concentrations and serum carotenoid concentrations are shown in Table 3. Each dietary carotenoid was significantly associated with its serum equivalent except for lycopene. Likewise, total VF intake was positively associated with all carotenoids except lycopene. Also, all categories of VF were positively associated with total serum carotenoids (with and without lycopene). Moreover, all categories of VF were positively associated with serum α-carotene, β-carotene and lutein. Besides orange vegetables, all categories of VF were also positively associated with serum β-cryptoxanthin while fruit, orange vegetables and dark green vegetables were significantly associated with serum zeaxanthin. None of the VF categories was correlated with serum lycopene concentrations. Metrics of adiposity (BMI, WC and FM) were mostly negatively associated with serum carotenoids except lycopene. Age was positively associated with serum concentrations of β-carotene and β-cryptoxanthin as well as with total serum carotenoids. TAG was negatively associated with all serum carotenoids except β-cryptoxanthin and lycopene. Total cholesterol was positively associated with all serum carotenoids expect α-carotene. LDL-cholesterol was positively associated with β-cryptoxanthin, lycopene and total serum carotenoids. Lastly, HDL-cholesterol was positively associated with all serum carotenoids except lycopene.

**Table 3.** Crude Spearman correlations of dietary carotenoids, vegetable and fruit intake, age, anthropometric measures and blood lipids concentrations with serum carotenoid concentrations (*n* 147)

	Serum carotenoid concentrations (μmol/l)							
	α-Carotene	β-Carotene	β-Cryptoxanthin	Lutein	Zeaxanthin	Lycopene	All carotenoids	All carotenoids without lycopene
Dietary carotenoids (μg)†	0·30***	0·35***	0·26**	0·28***	0·24**	0·10	0·17 a	0·40***
Vegetable and fruit intake (servings/d)								
Vegetables and fruit	0·43***	0·44***	0·26**	0·30***	0·24**	0·01	0·40***	0·44***
Fruit	0·23**	0·25**	0·19*	0·27***	0·22**	−0·02	0·24**	0·28***
Whole fruit	0·33***	0·32***	0·19*	0·28***	0·16	−0·09	0·30***	0·35***
Vegetables	0·41***	0·42***	0·20*	0·20*	0·15	0·05	0·38***	0·39***
Orange vegetables	0·32***	0·27***	0·11	0·24**	0·21*	−0·08	0·26**	0·30***
Dark green vegetables	0·28***	0·34***	0·29***	0·29***	0·23**	−0·08	0·30***	0·34***
Participant characteristics								
Age (years)	0·07	0·19*	0·22**	0·14	0·08	0·11	0·19*	0·20*
BMI (kg/m^2^)	−0·26**	−0·32***	−0·18*	−0·35***	−0·27**	−0·05	−0·33***	−0·35***
Waist circumference (cm)	−0·32***	−0·36***	−0·30***	−0·42***	−0·38***	0·02	−0·36***	−0·41***
Fat mass (kg)	−0·25**	−0·25**	−0·08	−0·27**	−0·25**	−0·13	−0·26**	−0·27**
TAG (mmol/l)	−0·37***	−0·34***	−0·13	−0·20*	−0·17*	0·15	−0·27***	−0·34***
Total cholesterol (mmol/l)	0·09	0·24**	0·34***	0·22**	0·24**	0·29***	0·29***	0·24**
LDL-cholesterol (mmol/l)	0·01	0·13	0·20*	0·06	0·12	0·30***	0·17*	0·11
HDL-cholesterol (mmol/l)	0·34***	0·41***	0·35***	0·45***	0·36***	−0·04	0·40***	0·45***

* *P* < 0·05, ** *P* < 0·01, *** *P* < 0·001.

† The variable ‘Dietary carotenoids’ represents the corresponding serum carotenoid listed at the top of the Table.

The stepwise regression model showed that VF intake, HDL-cholesterol, WC and age were the strongest predictors of total serum carotenoids without lycopene (Table 4). De-attenuation as well as adjustment of the univariate correlation for HDL-cholesterol, WC and age slightly increased the strength of the relationship between dietary carotenoid intake and serum carotenoid concentrations. Similarly, the adjustment strengthened the associations of total VF intake, vegetables as well as orange vegetables with total serum carotenoid concentrations without lycopene. However, the adjustment slightly decreased the associations of fruit, whole fruit as well as dark green vegetable intakes with total serum carotenoid concentrations without lycopene (Table 5).

**Table 4.** Variables included in the prediction model to determine total serum carotenoids without lycopene (significant at 0·15)

Variables*	Regression coefficient (*B*)	*P*	Model *P*	Model *R*^2^
Intercept	2·57	<0·01	<0·01	0·36
Total vegetable and fruit intake (servings/d)	0·15	<0·01		
HDL-cholesterol (mmol/l)	0·27	0·14		
Waist circumference (cm)	−0·03	<0·01		
Age (years)	0·01	0·02		

* Variables considered but not included in the prediction model were: TAG, total cholesterol, LDL-cholesterol, body weight, fat mass, BMI, sex, intake of supplements and total energy intake.

**Table 5.** De-attenuated† partial correlations between dietary intake and serum carotenoids adjusted for HDL-cholesterol, waist circumference and age (*n* 147)

	α-Carotene	β-Carotene	β-Cryptoxanthin	Lutein	Zeaxanthin	Lycopene	All carotenoids	All carotenoids without lycopene
Dietary carotenoids (μg)‡	0·41***	0·43***	0·34***	0·29**	0·24*	0·13	0·24*	0·49***
Vegetable and fruit intake (servings/d)								
Vegetables and fruit	0·45***	0·46***	0·24**	0·30***	0·23*	0·00	0·42***	0·48***
Fruit	0·22*	0·24**	0·16	0·27**	0·21*	−0·02	0·23**	0·27**
Whole fruit	0·30***	0·27**	0·11	0·23**	0·10	−0·01	0·24**	0·30***
Vegetables	0·48***	0·50***	0·22*	0·24**	0·18	0·04	0·45***	0·48***
Orange vegetables	0·39***	0·34***	0·13	0·32***	0·27**	−0·10	0·34***	0·40***
Dark green vegetables	0·26**	0·32***	0·26**	0·26**	0·19	−0·09	0·27**	0·31***

* *P* < 0·05, ** *P* < 0·01, *** *P* < 0·001.

† De-attenuated means adjusted for day-to-day variability.

‡ The variable ‘Dietary carotenoids’ represents the corresponding serum carotenoid listed at the top of the Table.

Approximately 80 % of respondents were classified in the same (40·8 %) or the adjacent (38·8 %) quartile of total VF intake and total serum carotenoid concentration without lycopene, while only 5·4 % of the respondents were classified in the opposite quartile. The κ statistic was 0·32 (Table 6). Moreover, there was a significant difference in total serum carotenoid concentration without lycopene of 43 % between the first (average of 3·2 servings of VF/d) and the fourth (average of 9·5 servings of VF/d) quartile of total VF intake. When total serum carotenoids was substituted by a composite variable created by the regression equation described in Table 4 (including VF, HDL-cholesterol, WC and age as predictors), the proportion of the participants classified in the same (44·9 %) or the adjacent (39·5 %) quartile reached 84·0 % and the proportion of participants classified in the opposite quartile was of 1·4 %.

**Table 6.** Cross-classification between quartiles of total vegetable and fruit intake and quartiles of total serum carotenoids (*n* 147)

	Same quartile	One quartile apart	Two quartiles apart	Opposite quartile
Proportion of respondents (%)	40·8	38·8	15·0	5·4
κ Statistic	0·32			

## Discussion

The present study was intended to validate the VF intake assessment from the R24W, a new web-based 24-h dietary recall, by using serum carotenoids as concentration biomarkers. We observed associations of moderate strength^(32)^ between dietary carotenoids and total serum carotenoids as well as between intake of VF and total serum carotenoids. Moreover, these associations were generally increased by the adjustment for relevant confounders.

Correlations between dietary and serum carotenoids observed in the present study are overall of similar magnitude than those observed in previous studies. Burrows *et al*.^(9)^ published a review of 142 validation studies using serum carotenoids as biomarkers. They reported average correlations between dietary carotenoids and serum carotenoids as follows: α-carotene, *r* 0·34 (95 % CI 0·31, 0·37); β-carotene, *r* 0·27 (95 % CI 0·25, 0·29); β-cryptoxanthin, *r* 0·38 (95 % CI 0·31, 0·37); lutein/zeaxanthin, *r* 0·29 (95 % CI 0·26, 0·33); lycopene, *r* 0·29 (95 % CI 0·26, 0·32). The only notable difference between results of the present study and those of Burrows *et al*.^(33)^ is that we did not find a significant association between dietary intake of lycopene and its serum concentration. However, this absence of association between dietary and serum lycopene is not uncommon. For example, Resnicow *et al*.^(30)^ also observed that dietary lycopene and serum lycopene were not significantly associated in a group of seventy-four adults who completed 3 d of 24-h recall. Furthermore, they came to the same conclusion with a larger group of 802 adults who completed a thirty-six-item FFQ.

In the present study, total VF intake was more strongly associated with α-carotene and β-carotene than with other serum carotenoids. In a recent study in which Burrows *et al*.^(33)^ validated a FFQ, α-carotene and β-carotene were also the strongest correlates of VF intake while lycopene was not associated with total VF intake. In fully controlled feeding trials, Couillard *et al*.^(18)^ reported a negative association between total VF intake and serum lycopene. Accordingly, lycopene is known to be a stronger correlate of tomatoes and tomato-based sauce intake than of the total amount of VF consumed^(12)^. We also found that the intake of vegetables was generally more strongly associated with total serum carotenoids than was the intake of fruit. Van Lee *et al*.^(34)^ noticed the same difference between vegetables and fruit using α-carotene, β-carotene, β-cryptoxanthin, lutein and zeaxanthin as biomarkers and two 24-h recalls as the dietary assessment method. As suggested by other researchers^(10,35)^, total VF intake in the present study was better predicted by total serum carotenoids than by individual carotenoids.

The association between reported VF intake and serum carotenoids was not expected to be perfect. Indeed, self-assessed dietary intake is associated with certain bias related to memory, estimation and desirability^(7,36)^. The concentration of carotenoids is also variable across the different types of VF and some food items not considered as a VF portion like ketchup contribute to the serum carotenoid concentration. However, using 4 d of 24-h dietary recall as a measure of the usual intake slightly attenuated the variability in the intake of dietary carotenoids as compared with only 1 d (variance ratios are presented in Supplementary Table S2). Furthermore, the bioavailability of carotenoids is limited because of multiple factors related to absorption and metabolism^(37)^. One demonstration of that is that even in controlled feeding trials, the associations between VF intake and serum carotenoids are not of high magnitude. In a study published by Souverein *et al*.^(21)^ in which data from twelve controlled feeding studies were used, it was found that unadjusted correlation coefficients between VF intake and serum carotenoids ranged between 0·08 and 0·29. Lastly, in the present study, blood measurements of carotenoids were conducted before the dietary intake assessment. The optimal delay between assessment of VF and measurement of carotenoids is difficult to establish. Indeed, Rock *et al*.^(38)^ proposed that the most common carotenoids stay in the serum for 12–33 d. Therefore, sampling carotenoids in the 12–33 d following dietary assessment would probably reflect more closely the reported intake of VF, which should result in stronger associations between VF intakes and carotenoid concentrations. We acknowledge that if our objective had been to specifically determine if serum carotenoids were good biomarkers of VF intake, it would then have been better to perform blood sampling after the administration of the dietary recalls. However, our main objective was to determine if our web-based 24-h dietary recall was able to adequately reflect the usual VF intake using carotenoids as concentration biomarkers. In such a context, we believe that performing blood samples before dietary assessment was an appropriate strategy. Finally, the fact that the magnitude of our correlations was similar to the one observed in other studies, including some performed in more controlled feeding conditions, suggests that dietary intakes measured in the present study (after blood sampling) are comparable with the intakes prior to blood sampling. This further suggests that our web-based 24-h dietary recall was able to adequately reflect the usual intake of VF intake.

To account for several factors influencing the association between VF intake and serum carotenoids, some authors have suggested adding selected confounders when examining this association. In a recent study conducted with data from fully controlled feeding trials, Allore *et al*.^(19)^ identified HDL-cholesterol, LDL-cholesterol and body weight as significant confounders of the association between the dietary intake of carotenoids and serum carotenoids. Similarly, the stepwise linear regression conducted in the present study showed that HDL-cholesterol and WC influenced the association between total VF intake and serum carotenoids. We also noticed significant associations between total serum carotenoids and other markers of adiposity such as FM and BMI as well as age, which corroborates observations from other studies^(33,39,40)^. Furthermore, the association between BMI and total serum carotenoids (*r* −0·33) was exactly the same as the one reported by Couillard *et al*.^(18)^ using data from fully controlled feeding trials. Also, as suggested by Allore *et al*.^(19)^, the difference in HDL-cholesterol concentration could be the most important factor explaining the difference in serum carotenoids between men and women. Indeed, in their cohort as well as in ours, women displayed higher concentrations of serum carotenoids even if they had lower intakes of dietary carotenoids than men. Interestingly, this sex difference was eliminated when the authors adjusted the serum carotenoid level for HDL-cholesterol concentration^(19)^.

In the present study, we found that the association between total VF intake and total serum carotenoids was slightly increased after adjustment for confounders. Similarly, Souverein *et al*.^(21)^ demonstrated in controlled feeding trials that, in a model adjusted for confounders, the correlation between intake of VF and serum carotenoids (including lycopene, folate and vitamin C) was strengthened from *r* 0·23 to *r* 0·77. This suggests that a correlation of low magnitude between VF intake and serum carotenoids does not necessarily reflect an inadequate reporting of food intake and can be explained to some extent by factors associated with the metabolism of serum carotenoids.

It is important to remember that the objective of the present study was to use serum carotenoids as concentration biomarkers of VF usual intake and not to predict the exact intake of total VF intake. Given the similarities between the relevant confounders identified in the present study and the ones identified in previous studies cited above as well as the strength of the associations between total VF intake and serum carotenoids concentration, we can be confident that the R24W assessed total VF intake with adequate relative validity.

Our cross-classification analysis reinforced observations from correlation analyses. First, we observed that almost 80 % of respondents were classified in the same or the adjacent quartile but also that less than 6 % were classified in the opposite quartiles. These observations were slightly better than those of Lai *et al*.^(41)^ for the validation of a FFQ in older adults where 68 % of respondent were classified in the same or the adjacent quartile using VF intake and serum carotenoids. We also noticed that levels of total serum carotenoids without lycopene among those with the highest VF intake (4th quartile) was 43 % higher than the serum levels of the smallest VF consumers (1st quartile). Lastly, the κ statistic was characteristic of an acceptable level of validity^(42)^.

It is important to mention that our sample was not fully representative of the Canadian population. Indeed, we recruited mostly well-educated French-speaking Caucasians. Acknowledging the possible racial influence on the associations between dietary and serum carotenoids^(43)^, this could limit the generalisability of the present results. Moreover, in our sample, the proportion of participants reporting more than five VF per d (62·7 % in women and 67·1 % in men) was higher than what has been observed in a survey of the Canadian population (36·9 % in women and 22·9 % in men^(44)^). The proportion of participants who reported using a multivitamin (20·4 %) was, however, comparable with the Canadian average (23·1 %^(45)^). Lastly, it could merit mentioning that our sample size calculation was based on energy intake and not on variation in carotenoid intakes, as the present study was conducted in the context of a larger validation study for which the under-reporting of energy intake was a key factor^(23)^. Nevertheless, knowing that our sample size was comparable with other studies using carotenoids as biomarkers of food intake^(9)^ and fulfils recommendations from the EURopean micronutrient RECommendations Aligned Network of Excellence (EURRECA)^(42)^, we are confident that it was large enough to identify significant associations.

### Conclusion

Overall, results of the present study support the appropriateness of the R24W to assess the dietary intake of VF on a group level and emphasise the importance of considering pertinent confounders when looking for the associations between VF intake and serum carotenoids.

## Supplementary material

For supplementary material accompanying this paper visit https://doi.org/10.1017/jns.2019.23. click here to view supplementary material
