# Supplementary material for: Associations between self-reported vegetable and fruit intake assessed with a new web-based 24-h dietary recall and serum carotenoids in free-living adults: a relative validation study
Source: J Nutr Sci. 2019 Aug 5;8:e26. doi: 10.1017/jns.2019.23 (PMC6683236; doi:10.1017/jns.2019.23)
Supplement: Supplementary file 1 [file S2048679019000235sup001.doc]

**Supplementary files**

**Supplementary Table S1. Coefficient of variation of each of the serum carotenoids using split samples**

|  | α-carotene | β-carotene | β-cryptoxanthin | Lutein | Zeaxanthin | Lycopene |
| --- | --- | --- | --- | --- | --- | --- |
| Coefficients of variation (%) | 6.64 | 3.68 | 4.26 | 4.03 | 5.85 | 10.03 |

**Supplementary Table S2. Variance ratio of dietary carotenoids according to the number of days of 24-hour dietary recall***

|  | α-carotene | β-carotene | β-cryptoxanthin | Lutein & Zeaxanthin | Lycopene |
| --- | --- | --- | --- | --- | --- |
| Variance ratio  (4-days vs 1-day) | 34% | 32% | 22% | 37% | 44% |
| Variance ratio  (3-days vs 1-day) | 37% | 34% | 28% | 44% | 43% |
| Variance ratio  (2-days vs 1-day) | 54% | 50% | 43% | 50% | 52% |

*Variance ratio are calculated as the average variance of 2, 3 or 4 24-hour dietary recalls divided by the variance of 1 24-hour dietary recall.
